# Generalization of Conditioned Contextual Anxiety and the Modulatory Effects of Anxiety Sensitivity

**DOI:** 10.1007/s13311-020-00831-8

**Published:** 2020-01-13

**Authors:** Marta Andreatta, Dorothea Neueder, Katharina Herzog, Hannah Genheimer, Miriam A. Schiele, Jürgen Deckert, Katharina Domschke, Andreas Reif, Matthias J. Wieser, Paul Pauli

**Affiliations:** 1 Department of Psychology (Biological Psychology, Clinical Psychology and Psychotherapy), University of Würzburg, Würzburg, Germany; 2 Department of Psychology, Educational Sciences, and Child Studies, Erasmus University Rotterdam, Burg. Oudlaan 50, 3062 DR Rotterdam, Netherlands; 3 Department of Psychiatry and Psychotherapy, Medical Center – University of Freiburg, Faculty of Medicine, University of Freiburg, Freiburg im Breisgau, Germany; 4 Department of Psychiatry, Psychosomatics, and Psychotherapy, Center of Mental Health, Würzburg, Germany; 5 Center of Mental Health, University of Würzburg, Würzburg, Germany; 6 Department of Psychiatry, Psychosomatic Medicine and Psychotherapy, University Hospital Frankfurt, Frankfurt am Main, Germany

**Keywords:** Context conditioning, Generalization processes, Startle response, Virtual reality, Anxiety sensitivity

## Abstract

Anxiety patients overgeneralize fear responses, possibly because they cannot distinguish between cues never been associated with a threat (i.e., safe) and threat-associated cues. However, as contexts and not cues are discussed as the relevant triggers for prolonged anxiety responses characterizing many anxiety disorders, we speculated that it is rather overgeneralization of contextual anxiety, which constitutes a risk factor for anxiety disorders. To this end, we investigated generalization of conditioned contextual anxiety and explored modulatory effects of anxiety sensitivity, a risk factor for anxiety disorders. Fifty-five participants underwent context conditioning in a virtual reality paradigm. On Day 1 (acquisition), participants received unpredictable mildly painful electric stimuli (unconditioned stimulus, US) in one virtual office (anxiety context, CTX+), but never in a second office (safety context, CTX−). Successful acquisition of conditioned anxiety was indicated by aversive ratings and defensive physiological responses (i.e., SCR) to CTX+ *vs* CTX−. On Day 2 (generalization), participants re-visited both the anxiety and the safety contexts plus three generalization contexts (G-CTX), which were gradually dissimilar to CTX+ (from 75 to 25%). Generalization of conditioned anxiety was evident for ratings, but less clear for physiological responses. The observed dissociation between generalization of verbal and physiological responses suggests that these responses depend on two distinct context representations, likely elemental and contextual representations. Importantly, anxiety sensitivity was positively correlated with the generalization of reported contextual anxiety. Thus, this study demonstrates generalization gradients for conditioned contextual anxiety and that anxiety sensitivity facilitates such generalization processes suggesting the importance of generalization of contextual anxiety for the development of anxiety disorders.

**Electronic supplementary material:**

The online version of this article (10.1007/s13311-020-00831-8) contains supplementary material, which is available to authorized users.

## Introduction

In order to increase survival, organisms have to learn which environmental stimuli predict threat or threat absence, which is safety [1]. Classical fear conditioning is an elegant and simple laboratory paradigm for modeling both threat and safety learning [2–4]. Briefly, during cue conditioning protocols, two stimuli are presented. One stimulus becomes the threat signal (conditioned stimulus, CS+) due to pairings with an aversive unconditioned stimulus (US, e.g., electric stimulation), whereas the other stimulus is never paired with the US and becomes the safety signal (CS−). As a consequence, the CS+ as compared to the CS− elicits fear responses as indicated by stronger freezing response in mice [5] and startle potentiation in rats [6] as well as increased aversive ratings, startle potentiation, and larger skin conductance response (SCR) in humans [7–9].

Anxiety patients frequently exhibit reduced discriminative responses to CS+ as startle responses are potentiated to the CS− [10–12], which is interpreted as deteriorated safety learning [13, 14]. Such reduced capacity in localizing safety may be the basis for an overgeneralization of fear responses [15–17]. Fear generalization refers to fear responses to stimuli, which resemble either physically or semantically the CS+, but have never been presented in association with the US. Focusing on physical similarity, healthy individuals show comparable fear responses, i.e., risk ratings, startle potentiation [18, 19], SCR [20], and amygdala activation [21], to CS+ and generalization stimuli if they are perceptually indiscriminable. In contrast, anxiety patients show fear responses to a broader range of stimuli indicating overgeneralization, possibly because they are less able than healthy individuals to discriminate generalization stimuli from the CS+ [18, 21–23].

Notably, the context, in which the learning takes place, is crucially implicated in the expression of conditioned fear [24, 25]. Moreover, the context itself may become associated with an aversive event and in consequence elicits defensive conditioned responses [26–29]. Importantly, contextual learning elicit anxiety rather than fear responses meaning that the former (i.e., anxiety) is a sustained response to a possible but not identified threat, whereas the latter (i.e., fear) is a phasic response to a concrete threat [30, 31]. Fear and anxiety are two distinct responses, which share only some neuronal mechanisms [32]. In other words, if the amygdala is involved in both learning, the hippocampus has a crucial role in anxiety learning [28, 29].

Such context conditioning differs in some crucial aspects from classical fear conditioning. First, the to-be-conditioned stimulus is rather complex, and studies in rodents outlined that single elements of the context (i.e., elemental representation) and/or the context as a whole (i.e., configural representation, 29) can be associated with the US. Second, the aversive event is unpredictable, and such unpredictability of threat induces a sustained fear response, i.e., anxiety mediated by the bed nucleus of the stria terminalis [BNST, 30, 31]. In a similar vein, rodents show strong anxiety-like responses such as freezing or startle potentiation within the context, i.e., the experimental cage, associated with the US, which is mediated by the activation of the BNST [28, 29, 32]. In order to translate experimental settings of animal studies more efficiently, virtual reality (VR) is often applied in human studies [26, 27, 33, 34]. In classical context conditioning studies using VR, participants have been guided into two virtual offices. In one office, the anxiety context (CTX+), the US is delivered unpredictably, whereas in the other office, the safety context (CTX−), no US is delivered. Successful learning is reflected by increased aversive ratings, startle potentiation, and larger skin conductance level (SCL) in the CTX+ as compared to the CTX− [26, 27]. Notably, context conditioning seems to better model learning mechanisms involved in panic disorder [PD, 13, 35].

Conceivably, conditioned contextual anxiety may become generalized as cued fear; however, this is much less investigated [15–17, 36]. To overcome this lack, we extended the abovementioned VR-context conditioning paradigm and examined anxiety responses to the anxiety and safety contexts plus to an additional virtual office, which was an equal mix of the anxiety and the safety context [37–39]. Interestingly, we found a dissociation between the verbal and the physiological responses of healthy individuals, i.e., they rated the generalization context (G-CTX) as aversive as the anxiety context but exhibited startle responses as in the safety context. We concluded that participants generalized conditioned anxiety on an explicit verbal level, but not on an implicit physiological level. The latter finding is in line with the literature on generalization of cued fear as these studies also revealed no generalization of conditioned physiological fear for stimuli, which share 50% of CS+ physical properties [18, 21, 23]. However, these cue studies were more elaborated as they were able to model generalization gradients on the basis of responses to a set of generalization stimuli ranging in similarity with the CS+ [18, 21, 23]. Such generalization studies still lack for context conditioning, and only one recent study in humans recreated such a generalization gradient by using five different long-lasting 2D pictures of a forest [36]. The pictures depicted the same forest, but during different seasons so that they shared some properties, but differed in others. Interestingly and in line with our studies [37–39], the authors found generalization of conditioned anxiety on the verbal level (i.e., ratings for the expectancy of the US), but not on the physiological level (i.e., startle response).

Therefore, the main goal of the current study was to investigate for the first time generalization of conditioned contextual anxiety by incorporating multiple generalization 3D contexts varying gradually in similarity to the anxiety context from 75% to 25%. Considering the role of anxious personality traits in generalization of conditioned fear [18, 21, 23], we additionally investigated the role of anxiety sensitivity (AS) as measured by the anxiety sensitivity index (ASI) in generalization of conditioned anxiety. We chose this personality trait because of two reasons. First, context conditioning is a good laboratory model for panic disorder [13, 35]. Second, anxiety sensitivity refers to the fear of bodily sensations and it can be a risk factor especially for panic disorder [40, 41]. Interestingly, AS seems to mediate over-estimation of fear in the insula and the dorsal anterior cingulate cortex [dACC, 42]. Therefore, it is conceivable that high anxiety sensitive individuals overgeneralize conditioned anxiety as well.

It is important to consider that fear and anxiety are two distinct responses, which share only some neuronal mechanisms [30, 32]. Accordingly, high anxious individuals show reduced discrimination between threat and safety signals [43–45, but see 46], paralleling the findings in anxiety patients [11]. On the contrary, acquisition of conditioned anxiety seems to be facilitated in high anxious individuals as quicker [47] and stronger [43] startle discrimination between CTX+ and CTX− suggests. Therefore, based on these findings on contextual learning, we hypothesized that the more anxiety sensitive participants are, the better they discriminate between the anxiety and the safety context. The role of trait anxiety on generalization of conditioned anxiety remains rarely investigated. In our previous study [37], we observed a slightly higher startle potentiation in the generalization context as compared to the safety context in high anxious individuals, but not in low anxious individuals. Therefore, in the current study, we expected greater generalization of conditioned anxiety in highly anxiety sensitive individuals.

## Methods

### Participants

Seventy-three healthy individuals participated voluntarily in the study and were recruited from the pool of the collaborative research center SFB TRR58.^1^ Exclusion criteria were age (before 18 and after 35 years of age), history of psychiatric or neurological disorders, high trait anxiety (T-score ≥ 60 calculated within gender and age, 48), actual use of psychoactive drugs, chronic pain, pregnancy, or color blindness. Students of psychology were excluded because of possible confounding factors due to their knowledge on conditioning. All nonpsychology student participants received 25 € for their participation.

Eight participants had to be excluded from the analysis because of low startle amplitude (overall mean amplitude below 5 μV, see “Methods”) and four additional participants were excluded, because no startle response was available in one or more condition. One participant was excluded because he/she suffered from migraine, one for technical problems, and four participants did not return for the second day of the experiment and therefore were excluded from all analyses. The final sample consisted of 55 participants (28 females; 27.69 years, *SD* = 6.59; for further descriptive statistics, see Table 1).

**Table 1:** Descriptive statistics

	M	SD	Min	Max
ASI	15.71	7.24	4	31
STAI X2	34.53	8.75	22	54
BDI	5.53	5.39	0	21
IPQ	− 2.15	11.99	− 29	22

All participants read and signed an informed consent approved by the ethics committee of the Medical Faculty of the University of Würzburg. They were informed about the possible side effects of VR, the loud noise, and that they will receive mild painful electric stimuli during the experiment. Participants were told to be attentive on the stimulus presentations but context and US contingency was not mentioned.

### Apparatus and Stimulus Material

#### Unconditioned Stimulus

*.* A constant current stimulator (Digitimer DS7A, Digitimer Ltd., Welwyn Garden City, UK) generated mildly painful electric stimuli (50 Hz, 200 ms) delivered through two electrodes to the dominant inner forearm triggered by the software CyberSession (VTplus GmbH, Würzburg, Germany; www.cybersession.info). The intensity was individually adjusted by means of two ascending and two descending series of electric stimuli. Participants had to rate each electric stimulus on a visual analogic scale (VAS) ranging from 0 (*no sensation at all*) to 10 (*very strong pain*) having the anchor for the threshold by 4 (*just noticeable pain*). The intensity of each shock was either increased or decreased by 0.5 mA. In order to determine the individual threshold, we considered the two first intensities rated as painful for the two ascending series, whereas for the two descending series were considered the last two intensities rated as painful. The pain threshold was increased by 30% in order to avoid habituation. After this procedure, the intensity of the electric stimuli was 2.11 mA (*SD* = 1.36) and was rated as painful (*M* = 5.35, *SD* = 1.19). Considering that participant returned in the laboratory 24 h later, we re-verified the intensity and its subjective painfulness at the beginning of the second day by applying the stimulus used on Day 1. In case a participant reported the US intensity of Day 1 as nonpainful (i.e., ≤ 3), we then increased the electric stimulation of 0.5 mA so often until participants indicated a painful intensity and used this new painful stimulus as aversive US for the second day. The intensity of the US on Day 2 (*M* = 2.26, *SD* = 1.35) was significantly higher (*F*_1,54_ = 13.01, *P* = 0.001, partial *η*^2^ = 0.194) than the US intensity on Day 1 and participants reported this more intense electric stimulus as painful (*M* = 5.02, *SD* = 1.24) as on Day 1 (*F*_1,54_ = 3.78, *P* = 0.057, partial *η*^2^ = 0.065). Importantly, US ratings were significantly higher than 4 (day 1: *t*_54_ = 8.39, *P* < 0.001; day 2: *t*_54_ = 6.09, *P* < 0.001) meaning that the US was reported painful on both days.

#### Contextual Stimuli (CTX)

We used the same VR environment described in [37]. Briefly, it consisted of virtual offices separated by a corridor and had the same floor plan, although they differed in the arrangement of the furniture. The aversive US was delivered in one office (anxiety context, CTX+), but never in the other office (safety context, CTX−). The offices were counter-balanced among participants. For the very first time, we created 3D and realistic stimuli, which shared physical properties in a gradual and proportional manner. A third virtual office was a mix of the other two and contained 50% of the furniture from one office and 50% of the furniture from the other office, equally distributed in the room. In addition, participants entered two other offices, which contained 75% of the furniture from one office and 25% of the furniture from the other office.

#### Startle Probes

The acoustic startle stimulus was a 103 dB burst of white noise presented for 50 ms binaurally via headphones (see "Procedure").

#### Ratings

Participants verbally rated the virtual offices after each experimental phase (see “Procedure”). A screenshot of a room was presented and participants were instructed to imagine being inside this virtual room. Below the screenshots, the following questions were asked consequently. For the valence ratings: “How pleasant vs. unpleasant was the office for you?”; for the arousal ratings: “How intense was your arousal in this office?”; for the anxiety rating: “How strong was your anxiety in this office?” and for the US expectancy ratings: “What is the probability that a painful electric stimulus was delivered in this office?”. A VAS ranging from zero until 100 was presented. Zero meant “negative,” “calm,” “low anxiety,” or “no association,” whereas 100 meant “positive,” “intense,” “strong anxiety,” and “perfect association,” respectively.

#### Questionnaires

Participants completed the German versions of several questionnaires (Table 1).

The Igroup Presence Questionnaire (IPQ, 49) measures the presence of participants in the VR. The Anxiety Sensitivity Index (ASI, 50) measures the individual anxiety sensitivity defined as fear of anxiety- or arousal related sensations such as increased heart rate. Both IPQ and ASI were filled in at the end of the second day. The State-Trait Anxiety Inventory (STAI, 48) consists of 20 items for the trait part and 20 items for the state part and measures the individual general anxiety. The Positive and Negative Affect Schedule (PANAS, 51) was used to determine current positive and negative mood on 10 items.

The STAI trait and state [48] as well as PANAS [51] were filled out at the beginning as well as at the end of the experiment on each day. Three 2 (time: begin, end) × 2 (day: acquisition, test) ANOVAs were calculated for the STAI state, for the positive scale and for the negative scale. We found that participants were more anxious (*M* = 36.17, *SD* = 7.53) and had less positive mood (*M* = 27.42, *SD* = 6.78) at the end of the experiment as compared to the beginning (STAI: *M* = 33.24, *SD* = 6.35, *F*_1,54_ = 15.40, *P* < 0.001, partial *η*^2^ = 0.222; PAS: *M* = 31.07, *SD* = 5.76, *F*(_1,54_ = 34.68, *P* < 0.001, partial *η*^2^ = 0.391). Moreover, participants had less negative mood on day 2 (*M* = 11.42, *SD* = 1.88) as compared to day 1 (*M* = 12.12, *SD* = 2.34, *F*_1,54_ = 16.77, *P* < 0.001, partial *η*^2^ = 0.237). No other effects were found (all *P* values > 0.085). Most likely, these changes are due to the delivery of aversive stimuli such as the painful electric shock (i.e., the US) and the white noise (i.e., the startle probe).

### Procedure

Participants came to the laboratory on two consecutive days at the same time of the day. On day 1, they signed the informed consent and completed a demographical questionnaire as well as the trait and the state parts of the STAI and the PANAS. Afterwards, the electrodes were attached (see “Data Reduction and Analysis”) and the pain threshold workup was conducted. Afterwards, participants underwent three phases and entered only the two to-be-conditioned offices, but not the three generalization offices (G-CTX).

During the *exploration phase*, participants navigated through two to-be-conditioned offices freely for 2 min by means of a joystick. No electric shock or startle probe was delivered.

In order to habituate the great initial reactivity of the startle response [52], seven startle probes were delivered every 9–17 s. Afterwards, the two identical acquisition phases *(Acquisition 1* and *Acquisition 2*) started. During these phases, participants were passively guided through the virtual offices on one of two prerecorded paths, played alternatively. Participants could still move their heads freely. All paths started from the corridor (intertrial interval, ITI) and entered one virtual room after 20 s for a duration of 140 s (one trial). Each acquisition phase consisted of six trials, i.e., three entries to each room. In one office (anxiety context, CTX+), participants received one to three USs in an unpredictable manner. No US was delivered in the other office (safety context, CTX−). Participants were asked to pay attention, but not mention was made about CTX-US contingency. Offices were counter-balanced among the participants. Additionally, during each trial, one to three startle probes were presented unpredictably during the contexts and four during the ITI (5–9 s after the start of the prerecorded path). Both USs and startle probes were never delivered during the first and the last 7 s of a room visit in order to prevent specific association between these aversive stimuli and the room’s doors. Moreover, the time intervals between two startle probes, or between two USs, or between a startle probe and an US were at least 10 s. After each phase, participants had to rate valence, arousal and anxiety of the office, and after the two acquisition phases, we asked about the expectancy of the US in the rooms.

Twenty-four hours later, participants returned in the laboratory and after having filled in the STAI state as the PANAS again, the electrodes were attached as on Day 1. Moreover, the intensity and painfulness of the US was verified by delivering one electric shock having the same intensity as on Day 1. In case participants reported the US as nonpainful (< 4 on the VAS), the intensity was increased to 0.5 mA until participants rated the electric shock as painful (for intensity and ratings of the US, see “Apparatus and Stimulus Material”). Then, we verified whether participants could recall the association between the contexts and the US 24 h later. Thus, participants had to rate valence, arousal and anxiety of both CTX+ and CTX− as well as their US expectancy. Afterwards, two identical test phases (*Generalization 1* and *Generalization 2*) started. Participants were passively guided into all five virtual rooms (i.e., CTX+, G75-CTX, G50-CTX, G25-CTX, and CTX−) on two different prerecorded paths. For each phase, each context was entered twice (altogether 20 trials, ten in each generalization test phase). In order to prevent extinction learning, two USs were unpredictably delivered during the last CTX+ trial of the Generalization 1. Furthermore, startle probes were delivered in each virtual room exactly as described for the acquisition phases. One to three startle probes were presented during the visit of each context and six during the ITI, in the same fashion as described above. As for day 1, after each phase, participants had to rate valence, arousal, and anxiety of the office, as well as the CTX-US expectancy.

### Data Reduction

Physiological responses were continuously recorded with a V-Amp 16 amplifier and Vision Recorder Software (Version 1.03.0004, BrainProducts Inc., Munich Germany). A sampling rate of 1000 Hz and a notch-filter at 50 Hz were applied. The offline analyses were conducted with the Brain Vision Analyzer (Version 2.0, BrainProducts Inc., Munich, Germany).

The eye-blink startle response was measured with electromyogram (EMG) from the *M. orbicularis oculi* with two 5 mm Ag/AgCl electrodes placed below the left eye following guidelines [52]. The EMG was offline filtered with a 28 Hz low-cutoff filter and a 400 Hz high-cutoff filter. Then, it was rectified and a moving average of 50 ms was applied for smoothing the signal. The signal was then segmented for each phase and virtual room from 50 ms before and 1 s after startle-probe onset. After the baseline correction (50 ms before probe onset), the startle responses were manually scored and trials with excessive baseline shifts (≥ 5 μV) were excluded from analysis. Startle amplitude was defined as the maximum peak between 20 and 120 ms after probe onset. Participants with a mean startle response < 5 μV were coded as nonresponders and excluded from the analysis (*N* = 8). In order to better detect interindividual differences, we decided to use raw data [53]. To this purpose, we calculated mean scores for each context during each phase, separately and subtracted the startle amplitudes of the ITIs from the startle amplitudes of the virtual offices.

Electro-dermal activity (EDA) was measured with two 8 mm Ag/AgCl electrodes, one fixed on the thenar and the other one on the hypothenar of the nondominant hand [54]. A 1 Hz high-cutoff filter was applied offline. Skin conductance levels (SCL) lower than 0.02 μS were scored as zero and then all SCLs were log10 transformed. SCL was calculated as mean EDA for the whole duration of the room’s visit (i.e., 125 s), except for the 10 s following the US. Alike the startle response, we calculated mean scores for each context (CTX+, G75-CTX, G50-CTX, G25-CTX, CTX−) separately for each phase (Acquisition 1, Acquisition 2, Generalization 1, Generalization 2). None of the participants had to be labeled as nonresponder for this response (i.e., mean SCL amplitude < 0.02 μS).

### Statistical Analysis

The statistical analysis was performed with the software IBM SPSS (Version 25, SPSS Inc.). In order to assure the comparability of the two to-be-conditioned context, we calculated three separated ANOVAs for each ratings after the habituation phase considering context (CTX+, CTX−) as within-subject factor. We first calculated ANOVAs separately for the 2 days for valence, arousal, anxiety, and US expectancy ratings as well as startle responses and SCL, respectively, in order to verify learning and generalization effects. ANOVAs for the acquisition day considered the within-subject factors context (CTX+, CTX−) and phase (Acquisition 1, Acquisition 2). ANOVAs for the generalization day had the within-subject factors context (CTX+, G75-CTX, G50-CTX, G25-CTX, CTX−) and phase (Generalization 1, Generalization 2). Notably, two additional analysis were conducted for investigating the generalization gradient of conditioned anxiety more in detail, that is the trend analysis [18]. Moreover and only for the ratings, we verified whether participant could discriminate between the context and therefore we calculated ANOVAs with within-subject factor context (CTX+, CTX−). Then for investigating the modulatory role of anxiety sensitivity, we calculated ANCOVAs separately for the two days considering the ASI sum score as covariate in order to investigate the modulatory influence of the anxiety sensitivity on anxiety learning and its generalization processes.^2^ In case of significant effects, we calculated Pearson correlation coefficients (two-tailed).

**Fig. 1:**
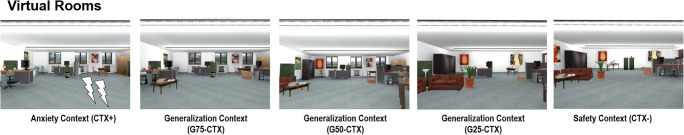
*Screenshots of the virtual contexts.* On day 1, participants were guided into either the anxiety context, in which they received one to three electric shocks (aversive US), or in the safety context, in which no US was delivered. On day 2, participants re-visited passively both CTX+ and CTX− as well as three additional contexts (generalization contexts or G-CTX), which gradually resembled the CTX+. Thus, G75-CTX consisted of 75% of the furniture from CTX+ and 25% of the furniture from CTX−; G50-CTX consisted of 50% of furniture from CTX+ and 50% from CTX−; and G25-CTX had 25% of furniture from CTX+ and 75% from CTX−

Partial *η*^2^ are indicated for effect size. In case of violation of the sphericity assumption, the Greenhouse-Geiser (GG-ɛ) correction was applied. The significant level was set at 0.05 and all post hoc tests were Bonferroni corrected (Fig. 1).

## Results

The results reported below are additionally depicted in violin plots in the Supplementary Material (Supplementary Fig 1 and 2, 55).

### Habituation Phase

#### Ratings

Valence (*F*_1,54_ = 0.99, *P* = 0.325, partial *η*^2^ = 0.018), arousal (*F*_1,54_ = 0.01, *P* = 0.938, partial *η*^2^ < 0.001), and anxiety (*F*_1,54_ = 1.66, *P* = 0.203, partial *η*^2^ = 0.030) ratings returned no significant effects indicating equal subjective properties of the two virtual offices before conditioning.

### Acquisition Phases

#### Ratings

**Fig. 2:**
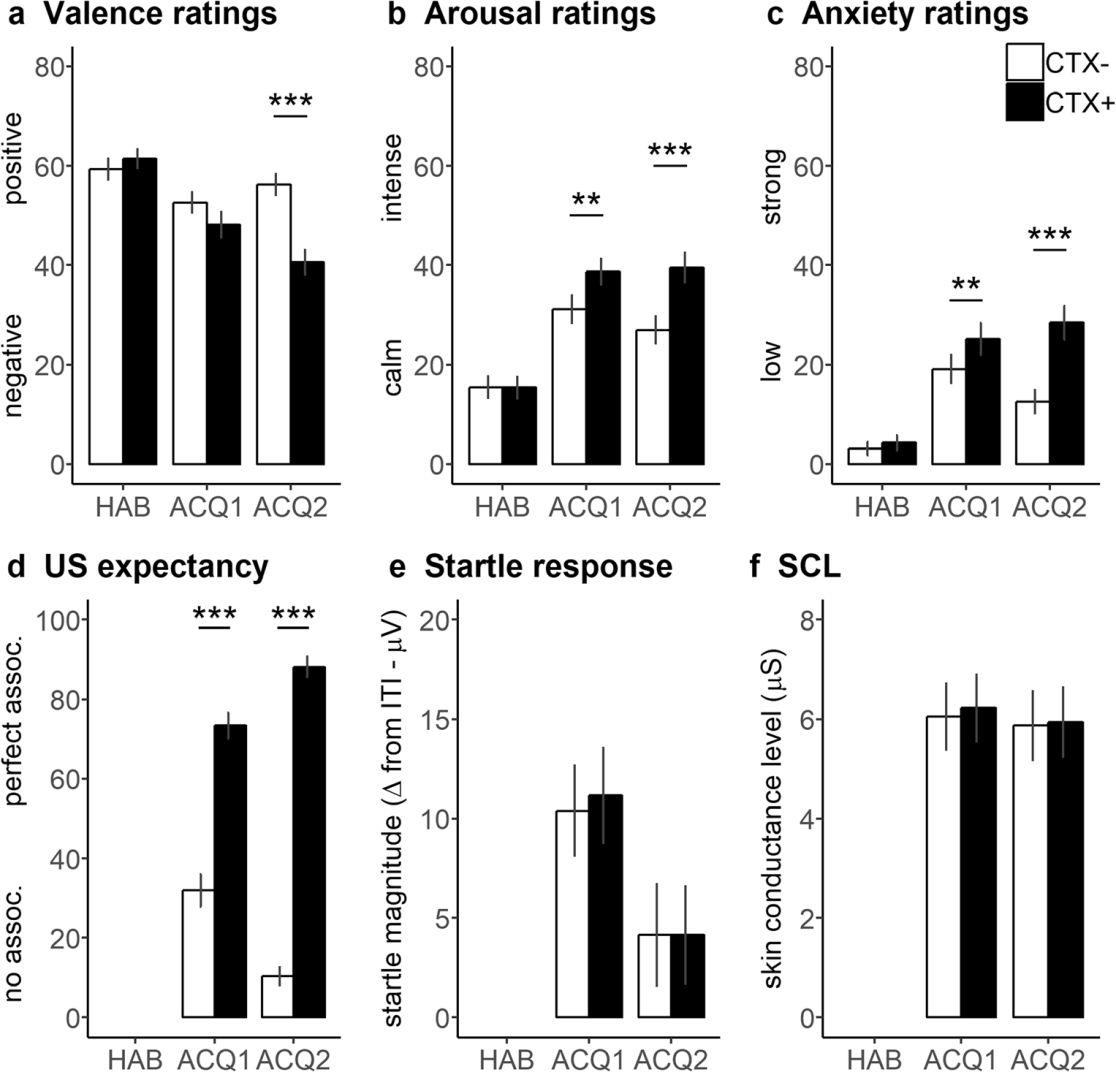
(**a**) Valence, (**b**) arousal, (**c**) anxiety, and (**d**) US expectancy ratings (with standard errors) after habituation (HAB), Acquisition 1 (ACQ1) and Acquisition 2 (ACQ2) as well as **e** startle responses and **f** skin conductance level to CTX+ (black bars) and CTX− (white bars). Discriminative responses to CTX+ vs. CTX− were evident for the ratings, and slightly for the physiological responses. **P < 0.01; ***P < 0.001

The main effect context turned out significant for all ratings meaning that CTX+ was rated significantly more negative (*F*_1,54_ = 11.61, *P* = 0.001, partial *η*^2^ = 0.177), arousing (*F*_1,54_ = 24.18, *P* < 0.001, partial *η*^2^ = 0.309), anxiogenic (*F*_1,54_ = 32.28, *P* < 0.001, partial *η*^2^ = 0.374) and associated with US (*F*_1,54_ = 222.18, *P* < 0.001, partial *η*^2^ = 0.804) than CTX−. No significant main effect for phase was found (all *P* values > 0.170). The interaction Context × Phase reached the significance level for valence (*F*_1,54_ = 20.24, *P* < 0.001, partial *η*^2^ = 0.273; Fig. 2a), arousal (*F*_1,54_ = 5.19, *P* = 0.027, partial *η*^2^ = 0.088; Fig. 2b), anxiety (*F*_1,54_ = 15.86, *P* < 0.001, partial *η*^2^ = 0.227; Fig. 2c), and US expectancy (*F*_1,54_ = 47.68, *P* < 0.001, partial *η*^2^ = 0.469; Fig. 2d) ratings.

Post hoc simple contrasts indicated that CTX+ was rated more arousing (*F*_1,54_ = 13.27, *P* = 0.001, partial *η*^*2*^ = 0.197) and anxiogenic (*F*_1,54_ = 8.42, *P* = 0.005, partial *η*^2^ = 0.135), but not more negative (*F*_1,54_ = 2.21, *P* = 0.143, partial *η*^2^ = 0.039) than CTX− after the first acquisition phase. Moreover, participants reported a significantly higher probability to receive the electric stimulus in CTX+ as compared to the CTX− (*F*_1,54_ = 56.91, *P* < 0.001, partial *η*^2^ = 0.513). After the second acquisition phase, CTX+ was rated more arousing (*F*_1,54_ = 24.35, *P* < 0.001, partial *η*^2^ = 0.311), anxiogenic (*F*_1,54_ = 41.29, *P* < 0.001, partial *η*^2^ = 0.433), and more negative (*F*_1,54_ = 21.29, *P* < 0.001, partial *η*^2^ = 0.283) than CTX−, and the US probability was significantly higher (*F*_1,54_ = 384.10, *P* < 0.001, partial *η*^2^ = 0.877).

#### Physiological Responses

For startle responses (*F*_1,54_ = 7.82, *P* = 0.007, partial *η*^2^ = 0.126; Fig. 2e), but not for SCL (*F*_1,54_ = 2.50, *P* = 0.120, partial *η*^2^ = 0.044), the main effect phase turned out significant, which may be related to habituation processes of these physiological responses. For the startle response, no further significant effects were observed (all *P* values > 0.781). Whereas for the SCL, we found a significant main effect context (*F*_1,54_ = 7.52, *P* = 0.008, partial *η*^2^ = 0.122; Fig. 2f), but not its interaction with phase (*F*_1,54_ = 2.29, *P* = 0.136, partial *η*^2^ = 0.041). In other words, participants showed stronger physiological arousal in CTX+ vs. CTX−.

#### Anxiety Sensitivity

Anxiety sensitivity significantly modulated the anxiety (Phase × ASI: *F*_1,53_ = 4.97, *P* = 0.030, partial *η*^2^ = 0.086: Context × Phase × ASI: *F*_1,53_ = 8.68, *P* = 0.005, partial *η*^2^ = 0.141), arousal ratings (Phase × ASI: *F*_1,53_ = 4.08, *P* = 0.048, partial *η*^2^ = 0.071) and US expectancy ratings (Context × ASI: *F*_1,53_ = 5.80, *P* = 0.020, partial *η*^2^ = 0.099), but no valence ratings (all *P* values > 0.108). Moreover, startle responses were also modulated by anxiety sensitivity (Context × ASI: *F*_1,53_ = 7.08, *P* = 0.010, partial *η*^2^ = 0.118), whereas for SCL, we found a significant interaction between phase and ASI (*F*_1,53_ = 4.96, *P* = 0.030, partial *η*^2^ = 0.085).

We then calculated correlations with the ASI scores for those effects involving the factor context. Thus, for the three-way interaction of the anxiety ratings, we calculated differential scores between CTX+ and CTX− for Acquisition 1 and Acquisition 2 respectively and then subtracted the differential score of Acquisition 2 the differential score of Acquisition 1 (Acq2[CTX+ minus CTX−] – Acq1[CTX+ minus CTX−]). When we correlated this final differential scores with anxiety sensitivity, we found a significant positive correlation (*r*(54) = 0.38, *P* = 0.005) meaning the more anxious participants were, the larger discriminative verbal responses were indicated after Acquisition 2 as compared to Acquisition 1. In order to disentangle the correlational effects, we separately correlated ASI with difference scores between CTX+ and CTX− after Acquisition 1 and after Acquisition 2 as well as difference scores between Acquisition 2 and Acquisition 1 separately for CTX+ and CTX−. We found (Bonferroni corrected *α* < 0.01) a positive correlation for CTX+ (*r*(54) = 0.39, *P* = 0.003), but not for CTX− (*r*(54) = 0.02, *P* = 0.859) meaning that the more anxiety sensitive participants were, the stronger anxiety ratings they reported in CTX+ after Acquisition 2 as compared to Acquisition 1. No significant correlations were found between ASI scores and CTX+/CTX− differential scores after both Acquisition 1 (*r*(54) = − 0.29, *P* = 0.031) and Acquisition 2 (*r*(54) = 0.13, *P* = 0.359).

For the two-way interactions, we first calculated the average between Acquisition 1 and Acquisition 2 of the US expectancy ratings as well as of the startle responses for CTX+ and CTX− respectively. Then, differential scores between CTX+ and CTX− were correlated with the ASI score. We found significant negative correlations between anxiety sensitivity and both differential US expectancy scores (*r*(54) = − 0.31, *P* = 0.020) as well as differential startle responses (*r*(54) = − 0.34, *P* = 0.010) indicating the more anxiety sensitive participants were, the less they discriminated between the two contexts.

### Recall of Conditioned Anxiety

#### Ratings

ANOVAs returned significant main effect context for all ratings (Table 2), which indicates that participants could remember the threatening context 24 h later. Thus, CTX+ was still rated more negative (*F*_1,54_ = 10.13, *P* = 0.002, partial *η*^2^ = 0.158), arousing (*F*_1,54_ = 30.79, *P* < 0.001, partial *η*^2^ = 0.363), and anxiogenic (*F*_1,54_ = 29.60, *P* < 0.001, partial *η*^2^ = 0.354) as compared to CTX− as well as participants expected US more in CTX+ than in CTX− (*F*_1,54_ = 182.16, *P* < 0.001, partial *η*^2^ = 0.771).

**Table 2:** Recall of conditioned anxiety. Ratings at the beginning of day 2, before generalization phase, on a scale ranging from 0 (no association) to 100 (perfect association)

	CTX+	CTX−
	M (*SD*)	M (*SD*)
Valence ratings (*SD*)	46.55 (18.58)	55.82 (16.49)
Arousal ratings (*SD*)	37.45 (23.23)	23.36 (19.32)
Anxiety ratings (*SD*)	27.15 (24.97)	13.64 (18.37)
US expectancy rating (*SD*)	86.58 (23.27)	14.93 (23.21)

#### Anxiety Sensitivity

Anxiety sensitivity did not influence the recall of conditioned anxiety. Thus, no significant effects of the covariate ASI were found for valence (all *P* values > 0.355), arousal (all *P* values > 0.152), anxiety (all *P* values > 0.117), and US expectancy ratings (all *P* values > 0.238).

### Generalization Test Phases

#### Ratings

**Fig. 3:**
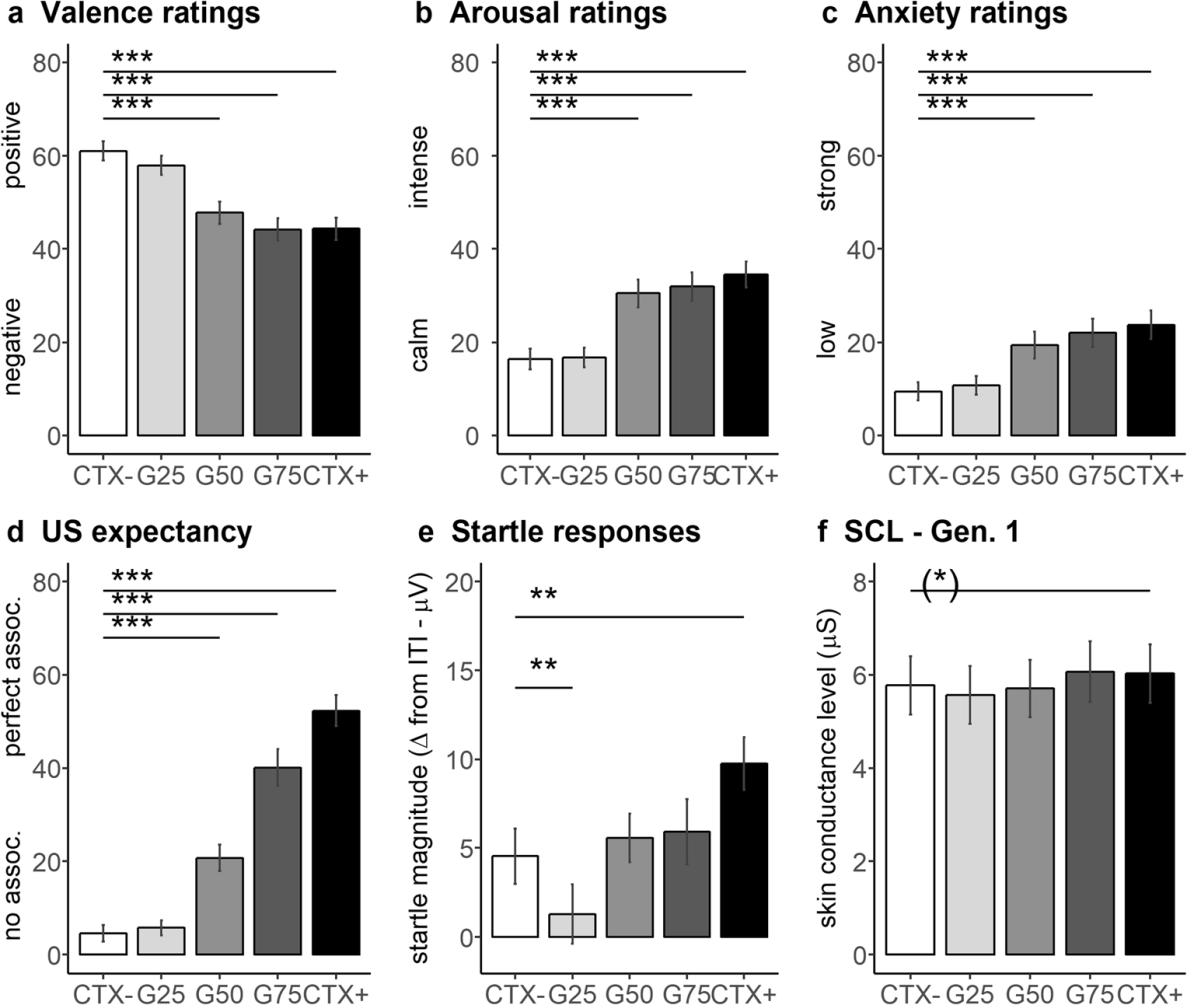
Responses (with standard errors) for (**a**) valence, (**b**) arousal, (**c**) anxiety, and (**d**) US expectancy ratings averaged between the two generalization phases, as well as (**e**) startle responses and (**f**) skin conductance levels to CXT− (white bars), G25-CTX (G25, light gray bars), G50-CTX (G50, gray bars), G75-CTX (G75, dark gray bars) and CXT+ (black bars). Participants generalized conditioned anxiety on the verbal level (i.e., ratings), but they generalized conditioned safety on the physiological level (i.e., startle responses and SCL). ^(^*^)^*P* < 0.05; **P < 0.01; ***P < 0.001

The main effects context (valence: *F*_4,216_ = 26.51, GG-ɛ = 0.461, *P* < 0.001, partial *η*^2^ = 0.329; arousal: *F*_4,216_ = 40.94, GG-ɛ = 0.616, *P* < 0.001, partial *η*^2^ = 0.431; anxiety: *F*_4,216_ = 26.97, GG-ɛ = 0.519, *P* < 0.001, partial *η*^2^ = 0.333; US expectancy: *F*_4,216_ = 85.76, GG-ɛ = 0.693, *P* < 0.001, partial *η*^2^ = 0.614; Fig. 3) and phase (valence: *F*_1,54_ = 4.19, *P* = 0.046, partial *η*^2^ = 0.072; arousal: *F*_1,54_ = 19.87, *P* < 0.001, partial *η*^2^ = 0.269; anxiety: *F*_1,54_ = 21.53, *P* < 0.001, partial *η*^2^ = 0.285; US expectancy: *F*_1,54_ = 57.33, *P* < 0.001, partial *η*^2^ = 0.515) turned out significant for all ratings. The interaction between context and phase reached the significance level for the US expectancy ratings (*F*_4,216_ = 18.98, GG-ɛ = 0.626, *P* < 0.001, partial *η*^2^ = 0.260; Table 3), but not for the other ratings (all *P* values > 0.380).

**Table 3:** US expectancy ratings after generalization phases

	Generalization 1	Generalization 2
CTX+ (*SD*)	67.71 (26.24)	37.00 (32.50)
G75-CTX (*SD*)	48.65 (32.58)	31.64 (30.51)
G50-CTX (*SD*)	28.20 (28.09)	13.27 (18.71)
G25-CTX (*SD*)	6.84 (12.77)	4.73 (14.09)
CTX− (*SD*)	5.84 (14.96)	3.27 (12.74)

As expected, no extinction learning was found, and after Bonferroni correction (*p* < 0.013), participants rated the CTX+ as more negative (*F*_1,54_ = 34.56, *P* < 0.001, partial *η*^2^ = 0.390), arousing (*F*_1,54_ = 68.86, *P* < 0.001, partial *η*^2^ = 0.560) and anxiogenic (*F*_1,54_ = 41.37, *P* < 0.001, partial *η*^2^ = 0.434) than the CTX−. Interestingly, participants generalized conditioned anxiety to G75-CTX and G50-CTX, because these offices were rated more negative (G75-CTX: *F*_1,54_ = 37.22, *P* < 0.001, partial *η*^2^ = 0.408; G50-CTX: *F*_1,54_ = 33.81, *P* < 0.001, partial *η*^2^ = 0.385), arousing (G75-CTX: *F*_1,54_ = 55.85, *P* < 0.001, partial *η*^2^ = 0.508; G50-CTX: *F*_1,54_ = 49.67, *P* < 0.001, partial *η*^2^ = 0.479), and anxiogenic (G75-CTX: *F*_1,54_ = 40.24, *P* < 0.001, partial *η*^2^ = 0.427; G50-CTX: *F*_1,54_ = 26.91, *P* < 0.001, partial *η*^2^ = 0.333) as compared to the CTX−. However, no differences between CTX− and G25-CTX could be discerned (all *P* values > 0.015). Post hoc simple contrasts for the US expectancy ratings revealed a similar pattern of responses after each generalization phase, namely participants expected the US more in the CTX+ than in the CTX− after Generalization 1 (*F*_1,54_ = 258.25, *P* < 0.001, partial *η*^2^ = 0.827) and after Generalization 2 (*F*_1,54_ = 61.81, *P* < 0.001, partial *η*^2^ = 0.534). Again, participants generalized conditioned anxiety and expected the US to occur more likely in the G75-CTX as well as in the G50-CTX than in the CTX− after both Generalization 1 (G75-CTX: *F*_1,54_ = 98.85, *P* < 0.001, partial *η*^2^ = 0.647; G50-CTX: *F*_1,54_ = 33.12, *P* < 0.001, partial *η*^2^ = 0.380) and Generalization 2 (G75-CTX: *F*_1,54_ = 52.57, *P* < 0.001, partial *η*^2^ = 0.493; G50-CTX: *F*_1,54_ = 16.45, *P* < 0.001, partial *η*^2^ = 0.234). As for the other ratings, no differences were observed between G25-CTX and CTX− (all *P* values > 0.252).

Analysis also revealed a significant linear trend for all ratings (valence: *F*_1,54_ = 35.69, *P* < 0.001, partial *η*^2^ = 0.398; arousal: *F*_1,54_ = 71.42, *P* < 0.001, partial η^2^ = 0.569; anxiety: *F*_1,54_ = 41.69, *P* < 0.001, partial *η*^2^ = 0.436; US expectancy: *F*_1,54_ = 204.61, *P* < 0.001, partial *η*^2^ = 0.791), whereas the quadratic effect was significant for valence (*F*_1,54_ = 11.40, *P* = 0.001, partial *η*^2^ = 0.174) and US expectancy (*F*_1,54_ = 9.33, *P* = 0.004, partial *η*^2^ = 0.147) ratings, but not for arousal (*F*_1,54_ = 3.21, *P* = 0.079, partial *η*^2^ = 0.056) or anxiety (*F*_1,54_ = 1.77, *P* = 0.189, partial *η*^2^ = 0.032).

#### Physiological Responses

The ANOVA for the startle response returned significant main effects context (*F*_4,216_ = 8.82, *P* < 0.001, partial *η*^2^ = 0.140; Fig. 3e) and phase (*F*_1,54_ = 4.19, *P* = 0.046, partial *η*^2^ = 0.072), but not their interaction (*F*_4,216_ = 1.59, GG-ɛ = 0.850, *P* = 0.186, partial *η*^2^ = 0.029). In contrast to the ratings and in line with previous studies [37, 38], on the automatic level of responses, participants did not generalize conditioned anxiety. Thus, startle responses were slightly potentiated in the CTX+ as compared to CTX− (*F*_1,54_ = 13.65, *P* = 0.001, partial *η*^2^ = 0.202), but not in G75-CTX (*F*_1,54_ = 0.71, *P* = 0.404, partial *η*^2^ = 0.013), G50-CTX (*F*_1,54_ = 0.51, *P* = 0.479, partial *η*^2^ = 0.009). Startle responses to G25-CTX were significantly more attenuated than in CTX− (*F*_1,54_ = 7.71, *P* = 0.008, partial *η*^2^ = 0.125).

The ANOVA for the SCL revealed significant main effect for context (*F*_4,216_ = 4.89, GG-*ɛ* = 0.598, *P* = 0.001, partial *η*^2^ = 0.083; Fig. 3f), but not for phase (*F*_1,54_ = 0.01, *P* = 0.931, partial *η*^2^ < 0.001), or their interaction reached the significance level (*F*_4,216_ = 0.32, *P* = 0.862, partial *η*^2^ = 0.006). Simple contrast to CTX− for the main effect context revealed marginally larger SCL to CTX+ as compared to CTX− (*F*_1,54_ = 6.32, *P* = 0.015, partial *η*^2^ = 0.105), but after Bonferroni correction (all *P* values < 0.013) no other contrasts turn out significant (all *P* values > 0.040).

In line with the ratings, the linear trend turned out to be significant for both physiological variables (startle responses: *F*_1,54_ = 16.52, *P* < 0.001, partial *η*^2^ = 0.234; SCL: *F*_1,54_ = 13.40, *P* = 0.001, partial *η*^2^ = 0.199), whereas the quadratic effect was significant for startle responses (*F*_1,54_ = 8.51, *P* = 0.005, partial *η*^2^ = 0.136), but not for SCL (*F*_1,54_ = 3.69, *P* = 0.060, partial *η*^2^ = 0.064).

#### Anxiety Sensitivity

As for the acquisition phases, no significant effects were found for anxiety sensitivity on valence (all *P* values > 0.061), arousal (all *P* values > 0.130), anxiety (all *P* values > 0.063), and US expectancy (all *P* values > 0.547) ratings. Moreover, for the startle response only the main effect of the covariate reached the significance level (*F*_1,53_ = 4.79, *P* = 0.033, partial *η*^2^ = 0.083), but no other effects were discerned (all *P* values > 0.127). The main effect for the covariate in the startle response returned a negative correlation (*r*(54) = − 0.29, *P* = 0.033) possibly due to the stronger startle responses to the baseline (i.e., ITI, see Supplemental Fig. 4c and also, 56).

**Fig. 4:**
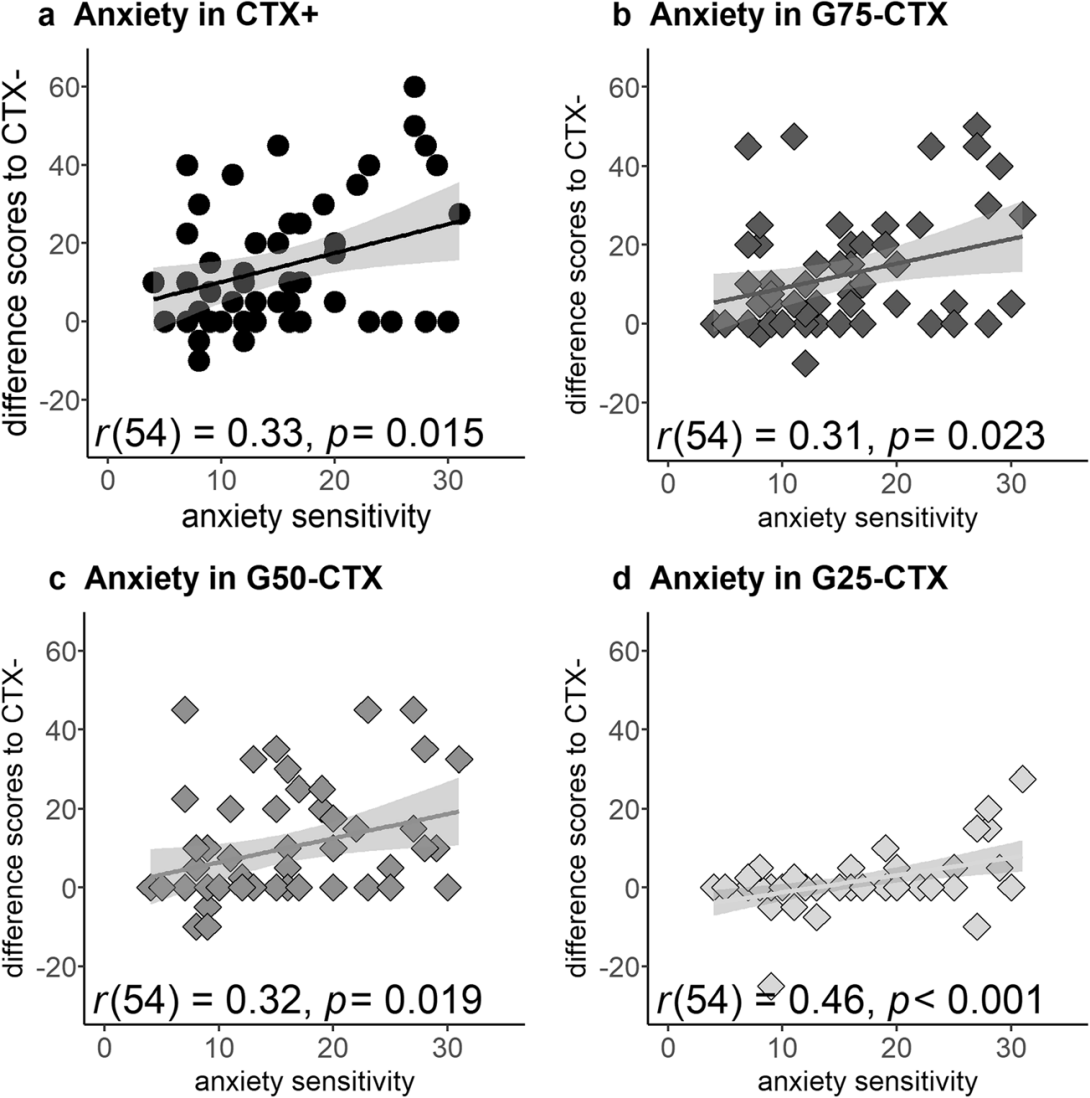
Correlations between anxiety sensitivity (measured by the Anxiety Sensitivity Index, ASI, x-axis) and differential anxiety ratings from the CTX− (y-axis). Positive correlations were found for **a** the CTX+ (black dots), **b** G75-CTX (G75, dark gray diamonds), **c** G50-CTX (G50, gray diamonds), **d** G25-CTX (G25, light gray diamonds). The more anxiety sensitive participants were, the more they generalized conditioned anxiety

Exploratively, we correlated ASI scores with the difference scores for the anxiety ratings between CTX− and the other contexts as the interaction Context × ASI turn out significant if no Greenhouse-Geiser correction was applied (*F*_4,212_ = 2.81, *P* = 0.027, partial *η*^2^ = 0.050). Strikingly, we found positive correlations between ASI scores and CTX+ (*r*(54) = 0.33, *P* = 0.015), G75-CTX (*r*(54) = 0.31, *P* = 0.023), G50-CTX (*r*(54) = 0.32, *P* = 0.019) as well as G25-CTX (*r*(54) = 0.46, *P* < 0.001), indicating that the higher the anxiety sensitivity, the more anxiogenic these contexts were rated (Fig. 4).

Moreover, ASI seemed to modulate SCL as the significant Phase × ASI (*F*_1,53_ = 4.89, *P* = 0.031, partial *η*^2^ = 0.085) and Context × Phase × ASI (*F*_4,212_ = 3.58, *P* = 0.008, partial *η*^2^ = 0.063) interactions suggested. However, when we tested the three-way interaction by correlating ASI scores with differential SCL scores between CTX− and the other contexts separately for Generalization 1 and Generalization 2, no significant correlation turn out (all *P* values > 0.112).

## Discussion

In this study, we investigated the generalization of conditioned anxiety and the modulatory role of individuals’ anxiety sensitivity. To this purpose, 55 healthy participants underwent a contextual learning protocol in virtual reality [26, 27] during which unpredictable aversive USs were delivered in one office (anxiety context), but never in a second office (safety context). Twenty-four hours later, participants returned to the laboratory. We firstly verified their memory trace for conditioned anxiety by means of ratings. Secondly, participants re-visited both anxiety and safety context as well as three additional virtual offices (generalization contexts). The physical properties of the generalization contexts gradually differed from the anxiety context, meaning one office (G75-CTX) shared 75% of the furniture with the anxiety context, one office (G50-CTX) displayed an equal mix of anxiety and safety context, and the third office (G25-CTX) shared only 25% of the furniture.

Stronger aversive ratings as well as physiological responses (i.e., SCR) for anxiety *vs* safety context indicate successful acquisition of conditioned anxiety, supporting previous findings in humans [27, 43, 47] as well as in animals [28, 29]. Given that this prerequisite for investigating generalization processes was fulfilled, we subsequently analyzed the responses to the generalization stimuli. Two main conclusions can be derived from the present results. First, the generalization of conditioned anxiety resembles the generalization of conditioned fear [18–20, 22]. Second, we found a clear positive generalization for verbal, but a weaker generalization for physiological conditioned anxiety, which parallels the findings in 2D paradigms [36].

Specifically, the trend analysis of our healthy sample revealed a significant linear effect for all variables, which suggests generalization of contextual anxiety and is in line with previous findings [36]. Importantly, a linear generalization gradient for conditioned anxiety in healthy individuals differs from the usually observed quadratic generalization effect for conditioned fear in healthy individuals [18, 23]. Although it is unclear why fear generalization is characterized by a quadratic trend, whereas anxiety generalization by a linear trend, it is possible that this difference is related to the distinct typologies of the measured responses that is fear *versus* anxiety [3, 30–32, 57] or to the complexity of the stimuli [28, 58]. Such discordance between fear and anxiety generalization processes should be systematically investigated in future studies in order to better understand which factors determine quadratic vs. linear trend.

Moreover, the verbal conditioned responses demonstrated that the G75-CTX and the G50-CTX were more anxiogenic than the CTX−, whereas the G25-CXT was rated as safe as the safety context. These results replicate previous findings [36–39] indicating that the generalization context with equal properties of anxiety and safety context was reported as threatening. Furthermore, we could extend these results as well as results on fear generalization [18–20, 22] by showing that the generalization context, which shared most properties with the anxiety context (i.e., G75-CTX), was rated as threatening as the anxiety context, whereas the generalization context, which shared least properties with the anxiety context (i.e., G25-CTX), did not result aversive.

The physiological conditioned responses to the generalization contexts, when directly compared to CTX−, did not reach the significance level suggesting no generalization effect, possibly related to poor statistical power. Alternatively and considering the anxiety generalization observed in the trend analysis, our sample could have been “too healthy” for generalization processes. Conceivably, high anxiety sensitive individuals would have shown both a linear generalization trend and startle potentiation to the generalization contexts as compared to the safety context. Although simple contrasts for physiological responses between generalization contexts and safety context is in contrast to the verbal reports, it is in line with previous studies in which the physiological conditioned anxiety was not generalized to the G50-CTX [36–39]. Notably, such safety generalization is also evident in the literature of cue conditioning, in which it has been found that the equal mix of the threat and safety signal does not elicit strong fear responses in healthy individuals [18–20, 22]. More unexpected are the safety-related physiological responses to the G75-CTX, because this context strongly resembled the anxiety context in number of elements as well as their position. Notably, this is also in contrast with the strong fear responses elicited by the generalization cues, which share most of the properties with the threatening signal.

Animal studies have shown that contextual learning consists of forming two representations [29, 59]. Thus, a context is the sum of its elements (i.e., elemental representation) as well as an environment as a whole (i.e., configural representation). Consequently, during contextual learning the US can be associated with the context as a whole, but also with its single elements. Considering our results, it is then conceivable that the ratings may have reflected the configural representation, whereas the physiological responses might have mirrored the elemental representation. This seems particularly true bearing in mind that at the end of each experimental phase participants were asked to recall the visited contexts in order to give their evaluations (i.e., by instructing them to recall the configural representation). In contrast, physiological responses were continuously recorded during the experimental phases. Although passively guided through the virtual offices, participants could freely move their head and therefore change their field of view. This might have resulted in a different set of US-associated elements of the anxiety context for each participant. Hence, during the generalization phase by randomly delivering startle probes, we may have not reached the equivalent number of US-associated elements in all participants in order to elicit physiological conditioned anxiety in G75-CTX. Supportively, two imaging studies in humans [60, 61] found distinct activation for elemental and configural representations for contextual learning involving the amygdala and the hippocampus, respectively. Importantly, in these studies participants’ field of view did not change as the authors used static pictures of two rooms and simply changed the disposition of the furniture.

Alternatively, such lack of generalization in physiological conditioned anxiety is also in line with configural theories [58]. Accordingly, using compound stimuli during associative learning determines two kinds of association, namely the US is predicted by the compound itself, but also by each single element of the compound. During test, two different results can be found. On the one hand, the single element entails as many predictive information about the US as the compound and consequently it is able to elicit the conditioned response (i.e., the CR is generalized). On the other hand, the single element is a distinct stimulus, different from the compound, which then is not able to elicit a CR. In our study, it is then possible that the single elements in the generalization contexts determined a new configuration, which was not enough informative about the US and consequently able to elicit conditioned physiological anxiety responses.

As mentioned above, conditioned anxiety was generalized only on the verbal level. The explorative analysis revealed that these responses were modulated by participants’ anxiety sensitivity (and trait anxiety, see Supplementary Material). In other words, the more anxiety sensitive individuals were, the better they discriminated between the safety context and all other contexts, that is higher anxiety ratings were reported in CTX+, G75-CTX, G50-CTX, and also in G25-CTX. Apparently, these findings do not fit with the “cue”-literature, which indicates the more anxious individuals are, the less they discriminate between the signals [18–20, 22].

Based on animal studies, cue and context conditionings model acquisition of fear and anxiety responses respectively, which are two distinct defensive responses [3, 30–32, 57]. Notably, such distinction is even more evident when considering clinical and sub-clinical samples that is anxious individuals show reduced discrimination between threat and safety signals [11, 43–45], but facilitated discrimination between threatening and safety situations [43, 47]. Therefore, our findings are in line with the distinction between fear and anxiety learning and support facilitated contextual discrimination in anxious individuals.

Interestingly, this was true for all generalization contexts, even for the G25-CTX, despite this context shared most of the properties with the safety context. Conceivably, the generalization contexts may have been quite ambiguous to the participants. As previously suggested [62], anxious individuals prefer “to be better safe than sorry”. Therefore, in ambiguous contexts they may have preferred to report stronger subjective anxiety due to the presence of few anxiety-related elements, despite most of the elements were safety-related. This is further supported by the shallow generalization gradient of conditioned fear in anxious individuals [15, 16, 18, 22, 23]. Thus, anxious individuals generalize their conditioned fear to a broader number of cues. However, anxiety patients did not generalize their fear to those stimuli, which only shared 15%/30% of their physical properties with CS+ [18, 22]. Strikingly, conditioned anxiety was generalized to a context, which only shared 25% of its physical properties with the CTX+ highlighting distinct generalization mechanisms. Therefore, it seems plausible that the very few anxiety-related elements in G25-CTX were enough to elicit strong defensive responses in anxious individuals.

As mentioned in the introduction, VR is an ergonomic tool, which allows to recreate reality resembling environments. Importantly, patients are more prone to be confronted with a feared or a traumatic situation in VR than in the real world [63, 64] as they know they remain in a safe environment (i.e., the laboratory or the clinic). During exposure therapy patients have first to image the aversive situation in order to learn to control their emotional reactions, and only in a second moment they are exposed to a real situation [65]. VR allows to overcome the abstractness of the imagination and patients with fear of height are asked to walk up the stairs of a virtual lookout [63]. Furthermore, VR allows to expose patients to aversive situations, which highly resemble the traumatic situation and which would not be otherwise ethically permitted, although remaining in a safety environment [64]. Referring to our study, we recreated a daily situation (i.e., offices) and demonstrated that anxiety sensitivity might modulate the responses to safety situations (i.e., the generalization contexts), which physically resembled the aversive situation (i.e., the anxiety context). It would be therefore interesting to extend our findings to feared or traumatic situations in order to investigate generalization processes in more disorder-specific situations and to better disentangle the elemental and configurational mechanisms involved in the maintenance of anxiety disorders or post-traumatic stress disorders (PTDS). Considering the efficacy of virtual exposure therapy (VRET, 66, 67), disentangling elemental and configural processes underlying generalization of conditioned anxiety allows us to specifically target pathological mechanisms during a therapy.

Some limitations of this study should be mentioned. We did not observe generalization of the physiological conditioned responses. Although it is possible that participants do not generalize conditioned anxiety on the physiological level (see also, 22), in future studies, it may be interesting to control the elemental representation of the context. In other words, to verify for each participant which element is associated with the US in order to better control these elements during the generalization phase. We observed a modulatory role of anxiety sensitivity on anxiety generalization processes. However, it would be interesting to apply this paradigm to a clinical population in order to verify whether patients with anxiety disorders show facilitated discrimination between safety and anxiety context in the same way as sub-clinically anxious individuals. Lastly, it would have been interesting to let participants freely move through the contexts (i.e., anxiety, safety, and generalization context) in order to observe their “spontaneous” behavior (e.g., covered distance).

## Conclusion

In sum, we discerned generalization of conditioned anxiety, which was modulated by the individuals’ anxiety sensitivity. Importantly, the anxiety generalization gradient shared some properties with the generalization of conditioned fear, but it also differentiated in many others aspects. First, healthy individuals showed a linear trend of anxiety generalization. Second, on the physiological level, conditioned anxiety was weakly generalized, which may be related to the complexity of the learning (elemental *vs* configural representations of the context). Third, anxiety sensitivity facilitated discrimination between safety context and all generalization contexts. Together, our results point to similar processes in anxiety and fear generalization, with subtle differences possibly due to more complex cognitive processes involved in context learning.

## Electronic Supplementary Material

ESM 1 (DOCX 4202 kb)

ESM 2 (PDF 434 kb)

## Footnotes

1 Participants were genotyped for the gene rs2180619 of the cannabinoid receptor type 1 (*CB-1*). This selection was done for the dissertation thesis of D. Neueder. The data has not been published.

2 Considering that most studies on cue conditioning considered trait anxiety measured by the STAI (Laux, Glanzmann, Schaffner, Spielberger: **Das State-Trait Angstinventar.** Weinheim: Beltz Test.; 1981), we additionally calculated ANCOVAs considering the STAI sum as covariate, exactly as we calculated for the ASI. Results are reported in the Supplementary Material.
