# Supplementary material for: Generalization of Conditioned Contextual Anxiety and the Modulatory Effects of Anxiety Sensitivity
Source: Neurotherapeutics. 2020 Jan 13;17(3):1239–52. doi: 10.1007/s13311-020-00831-8 (PMC7609477; doi:10.1007/s13311-020-00831-8)
Supplement: Supplementary file 1 — (DOCX 4202 kb) [file 13311_2020_831_MOESM1_ESM.docx]

**Generalization of conditioned contextual anxiety and the modulatory effects of anxiety sensitivity**

Marta Andreatta^1^, Dorothea Neueder^1^, Katharina Herzog^1^, Hannah Genheimer^1^, Miriam A. Schiele^2^, Jürgen Deckert^3, 6^, Katharina Domschke^2,3^, Andreas Reif^4^, Matthias J. Wieser^1,5^, Paul Pauli^1, 6^


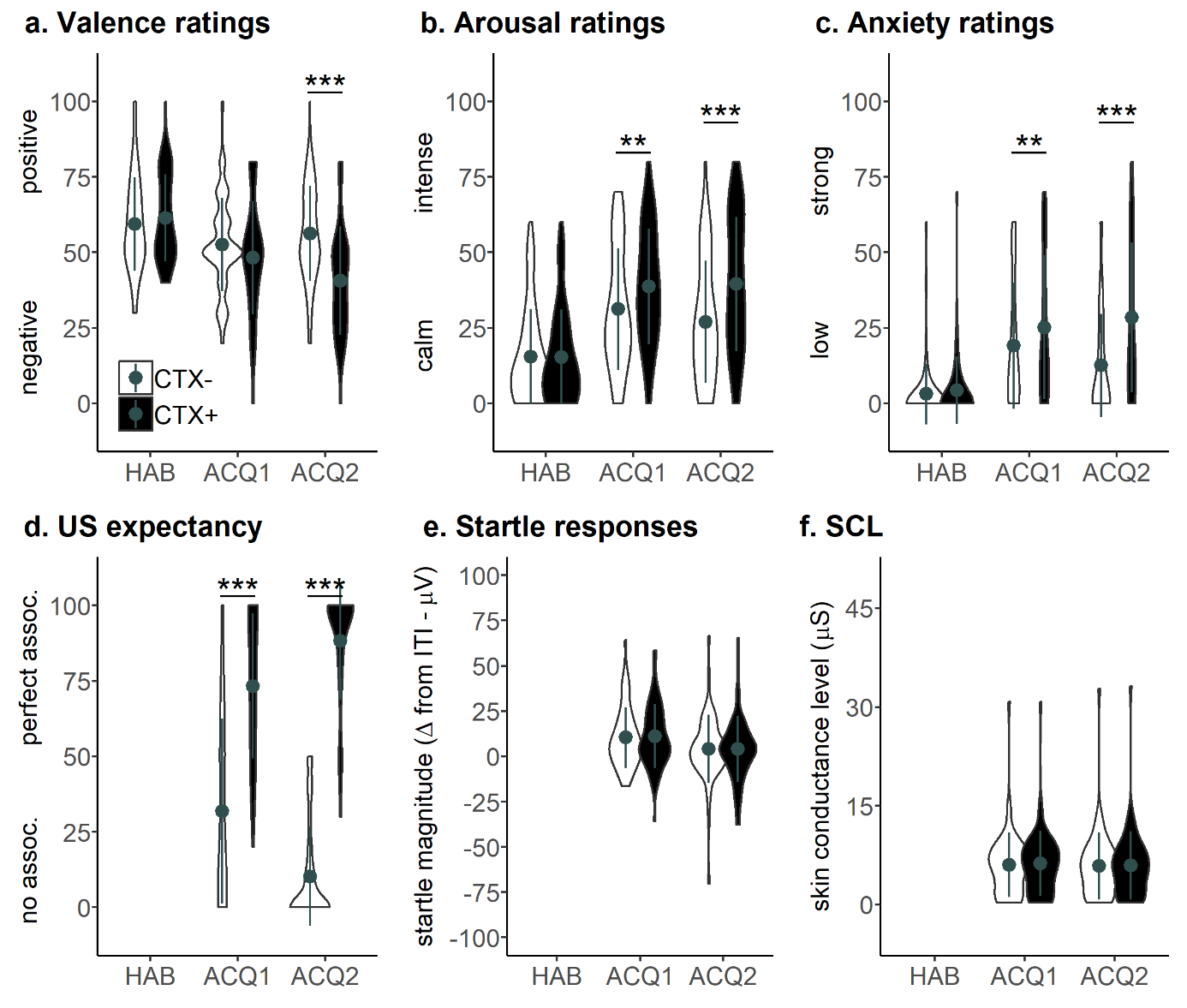


**Supplementary Fig. 1**. (**a.**) Valence, (**b.**) arousal, (**c.**) anxiety and (**d.**) US expectancy ratings as well as (**e.**) startle responses and (**f.**) skin conductance level (SCL) after habituation (HAB), Acquisition 1 (ACQ1) and Acquisition 2 (ACQ2) to CTX+ (black violins) and CTX- (white violins). Dots indicate means with standard deviations. Discriminative responses to CTX+ vs. CTX- were evident for the ratings, and slightly for the physiological responses. ** *P* < 0.01; *** *P* < 0.001


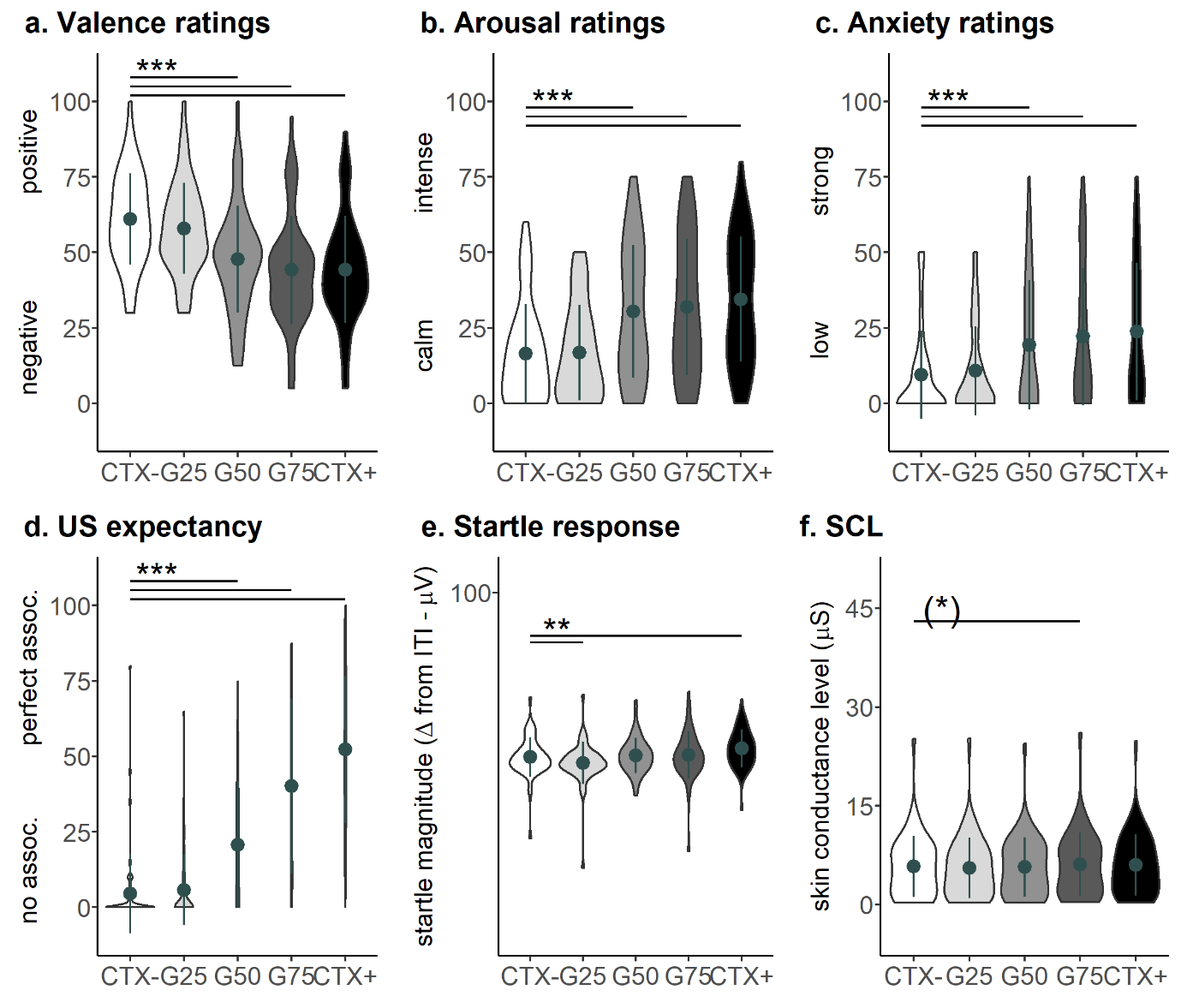


**Supplementary Fig. 2**. Responses for (**a.**) valence, (**b.**) arousal, (**c.**) anxiety and (**d.**) US expectancy ratings averaged across the two generalization phases, as well as (**e.**) startle responses and (**f.**) skin conductance levels (SCL) to CXT- (white violins), G25-CTX (G25, light grey violins), G50-CTX (G50, grey violins), G75-CTX (G75, dark grey violins) and CXT+ (black violins). Dots in the middle depict means with *sd*. Participants generalized conditioned anxiety on the verbal level (i.e., ratings), but they generalized conditioned safety on the physiological level (i.e., startle responses and SCL). ^(^*^)^ *P* < 0.05; ** *P* < 0.01; *** *P* < 0.001


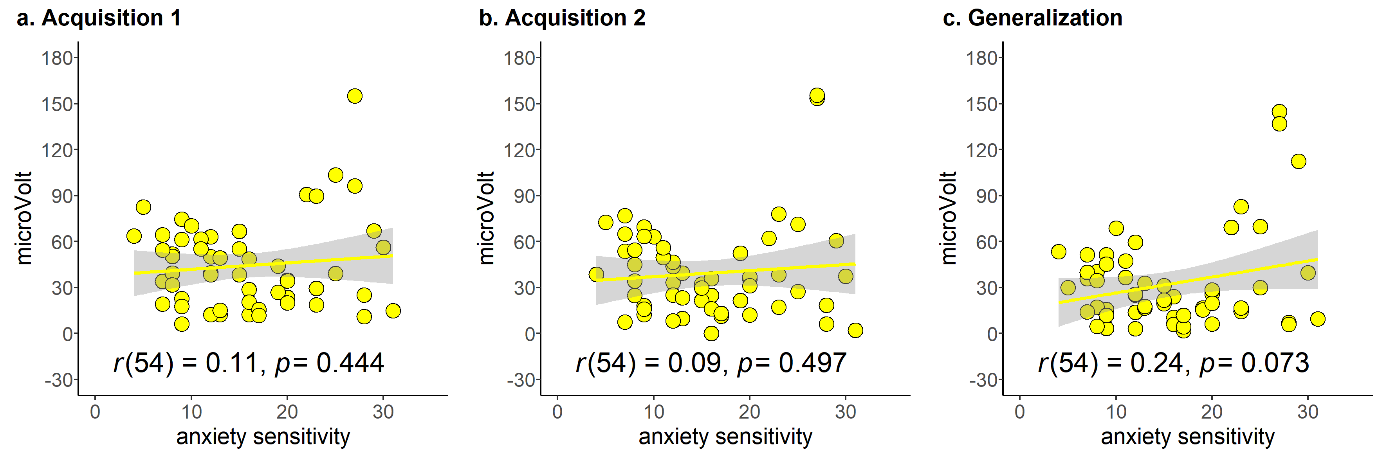


**Supplementary Fig. 4**. Correlations between anxiety sensitivity (on the x-axis) and the startle responses during the ITI (on the y-axis) during (**a.**) Acquisition 1, (**b.**) Acquisition 2, and (c**.**) generalization phases. As mentioned in the discussion, responses to the ITI during the generalization phase were slightly modulated by anxiety sensitivity meaning the more anxious participants were, the stronger baseline startle responses were observed.

**Supplementary Fig. 3**. Startle responses (with standard errors) during (**a.**) acquisition and (**b.**) generalization phases, respectively on Day 1 and on Day 2. Here, we separately depicted the startle responses during inter-trial interval (ITI, yellow bars). As indicated in the main text, black bars indicate startle responses to CTX+, white bars startle responses to CTX- and the gradual grey bars, the startle responses to the generalization contexts.


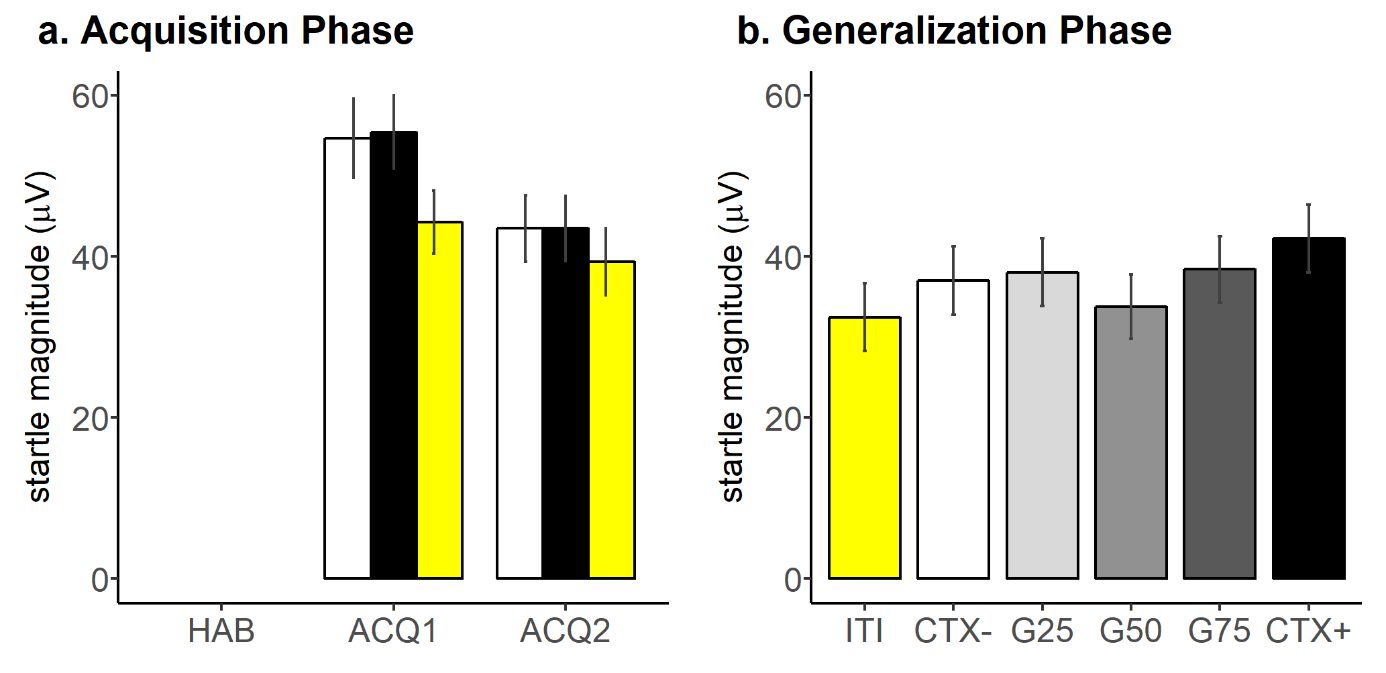


**Supplementary analyses**

*Covariate STAI X2.* Alike for the ASI, ANCOVAs separately for the two days were calculated considering the STAI X2 sum score as covariate. In case of significance effects, we calculated Pearson correlation (two-tailed).

**Supplementary results**

**Acquisition phase**

No significant effects for the covariate STAI X2 were found for both the ratings (valence: all *P* values > 0.130; arousal: all *P* values > 0.124; anxiety: all *P* values > 0.140; US expectancy: all *P* values > 0.254) and the physiological responses (startle responses: all *P* values > 0.178; SCL: all *P* values > 0.255).

**Recall of conditioned anxiety**

The covariate interacted significantly with the factor context for both arousal ratings (*F*_1,54_ = 5.21, *P* = 0.026, partial η^2^ = 0.090), but not for valence (*F*_1,54_ = 3.64, *P* = 0.062, partial η^2^ = 0.064), anxiety (*F*_1,54_ = 3.80, *P* = 0.056, partial η^2^ = 0.067) and US expectancy (*F*_1,54_ = 0.73, *P* = 0.396, partial η^2^ = 0.014) ratings. No other effects were revealed (all *P* values > 0.056).

In order to investigate the effect of the covariate on the arousal, we then calculated differential scores between CTX+ and CTX- and correlated these with the STAI X2 scores. The more anxious participants were, the stronger they differentiated between the two contexts 24 hours after learning (arousal: *r*(54) = 0.30, *P* = 0.026; Supp. Fig. 5).


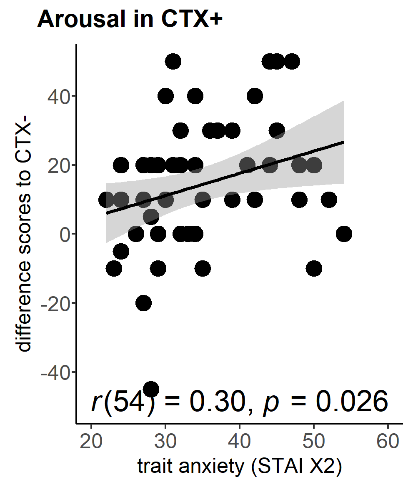


**Supplementary Fig. 5**. Correlations between trait anxiety (STAI-2, x-axis) and differential arousal ratings from the CTX- (y-axis) at the beginning of Day 2. Positive correlations were found meaning the more anxious participants are, the better they discriminate between anxiety and safety context.

**Generalization phase**

The trait anxiety of the participants modulated significantly the anxiety (*F*_4,212_ = 5.51, GG-ε = 0.541, *P* = 0.004, partial η^2^ = 0.094; Supp. Fig. 6) ratings to the five contexts, but no other dependent variables (valence: all *P* values > 0.174; arousal: all *P* values > 0.085; US expectancy: all *P* values > 0.151; startle responses: all *P* values > 0.189; SCL: all *P* values > 0.241) [1].

**Supplementary Fig. 6**. Correlations between trait anxiety (STAI-2, x-axis) and differential anxiety ratings from the CTX- (y-axis) averaged for the two generalization phases. Positive correlations were found for CTX+ as well as for all generalization contexts meaning the more anxious participants are, the more anxiougenic was rated the context.


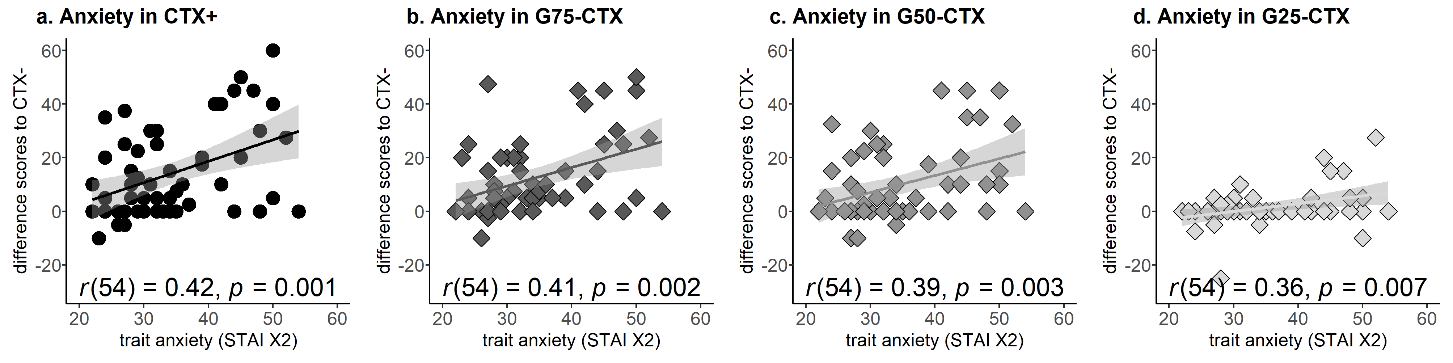


We then followed the interaction between the covariate and the context, by calculating differential scores between CTX- and the other contexts (i.e., CTX+, G75-CTX, G50-CTX, G25-CTX) and correlating these scores with the sum STAI X2 score. STAI scores positively correlated with the differential scores of CTX+ (*r*(54) = 0.42, *P* = 0.001), G75-CTX (*r*(54) = 0.41, *P* = 0.002), G50-CTX (*r*(54) = 0.39, *P* = 0.003), or G25-CTX (*r*(54) = 0.36, *P* = 0.007).

**Pilot Study**

In order to assure a proportional distribution of the furniture in the three generalization contexts, we conducted several pilot studies. The pilot studies for the office G50-CTX have been already where else reported [2]. For the offices G75-CTX and G25-CTX two additional pilot studies were run.

*Participants*. Sixteen and eleven volunteers participated at the two pilot studies, respectively. For the first pilot study, the mean age of the 16 participants was 22.75 years (*SD* = 3.34, range: 19-30 years), and for the second pilot study the mean age of the eleven participants was 27.82 years (*SD* = 9.71, range: 19-51 years).


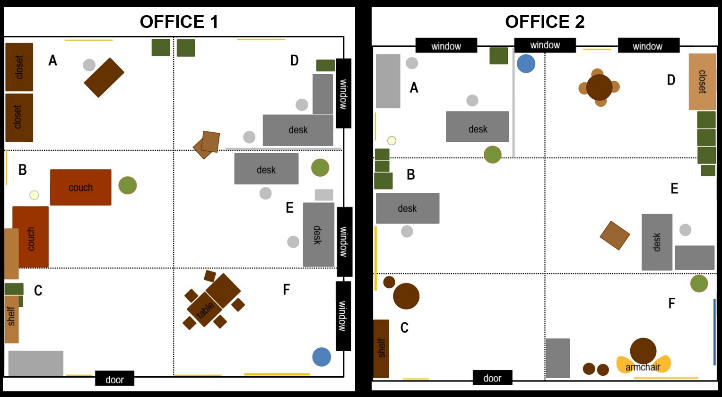


**Supplementary Fig. 7**. Sketch of the two offices (i.e., Office 1 and Office 2), which worked as either anxiety context or safety context during acquisition phase of the experiment. The offices were divided in six equal sections and from each section the proportional amount of furniture were re-located in the generalization offices (75% vs. 50% vs. 25%).

*Procedure*. In order to have the correct amount of furniture for the anxiety and the safety context in the generalization contexts, we divided each office into six equal rectangular sections (Supplementary Fig. 7). In each sections, 75% of the furniture was taken from Office 1 and 25% was taken from Office 2 or the other way around (i.e., 75% from Office 2 and 25% from Office 1). Importantly, the furniture had the same location as in the original office. Firstly, participants explored the five rooms freely by means of a joystick for ca. 2 min. Secondly, participants were passively guided into the five rooms for ca. 2 min. At the end of this exploration phases, screenshots of all five rooms were then rated in regard to the valence (“how negative or positive was this room?”; scale ranging from 0 – negative – until 100 – positive) and arousal (“how arousing was this room?”; scale ranging from 0 – calm – until 100 – intense). Moreover and importantly, similarity ratings were asked for both the offices as whole and for each rectangle. For these ratings, either the screenshot of the Office 1 or the screenshot of the Office 2 was presented in compound with the screenshot of either Office 4 or Office 5 (i.e., four combinations: Office 1/Office4, Office 1/Office 5, Office 2/Office4, Office2/Office5). Below the screenshots, the question “how similar are these two rooms?” was written and participants gave their rating based on a visual analog scale (VAS) ranging from 0 (not similar at all) to 100 (identical) was presented.

*Analysis*. Considering that Office 5 should have been 75% similar to Office 1, but 25% similar to Office 2, we subtracted 75 from the Office1/Office5 ratings and 25 from the Office1/Office4 ratings. The other way around was calculated for Office 2 as Office 5 should be 25% similar and Office 4 75% similar. The same difference scores were calculated for each single rectangle. The differential scores should even out the percentage of similarity and therefore no difference are expected between Office 4 and Office 5.

Additionally, we controlled that the similarity of the two new offices (i.e., Office 4 and Office 5) was comparably to the Office 3 (i.e., the office sharing 50% of the furniture from Office 1 and Office 2). To this purpose, we subtracted 55 from Office3/Office4 ratings and 35 from Office3/Office5 as Office 4 should be 35% similar, while Office 5 should be 55%.

We then calculated *t*-tests of the difference scores for Office1/Office4 and Office1/Office5 as well as for Office2/Office4 and Office2/Office5 separately. In addition, we compared similarity ratings between Office 4 or Office 5 and Office 3.

*Results*. Comparisons returned that similarity ratings for Office 4 were significantly different between Office 1 and Office 2 (*t*_15_ = 7.04, *P* < 0.001) as well as similarity scores for Office 5 between Office 1 and Office 2 (*t*_15_ = 2.27, *P* = 0.039) suggesting that the percentage of similarity was not correctly reached (Supplementary Fig. 8a). Moreover, similarity ratings for Office 4 and Office 5 vs. Office 3 differed significantly (*t*_15_ = 4.16, *P* = 0.001). We then verified whether such inequalities in the similarity were driven from a single section of the offices. For each section, six *t*-test comparisons were calculated (Bonferroni corrected α < 0.008) for either Office 4 or Office 5 between Office 1 and Office 2. For Office 4, section D (*t*_15_ = 3.47, *P* = 0.003) and section E (*t*_15_ = 3.15, *P* = 0.007) resulted significantly unequal. While, sections A (*t*_15_ = 4.73, *P* < 0.001) and D (*t*_15_

= 6.85, *P* < 0.001) were unequal for Office 5 between Office 1 and Office 2. Moreover, section A (*t*_15_ = 3.47, *P* = 0.003) and section E (*t*_15_ = 3.62, *P* = 0.003) of the two new offices were significantly different from the sections in Office 3. All other comparisons did not reach the significance level (all *p*s > 0.039).

For this reason, we re-arranged these sections and conducted a second pilot study, which had the same procedure as described above and the similarity ratings were only asked for the changed sections. No significant difference in similarity ratings between Office 1 and Office 2 were found in section A (*t*_10_ = 0.22, *P* = 0.833) and E (*t*_10_ = 0.75, *P* = 0.472) of Office 5 as well as in section D (*t*_10_ = 1.62, *P* = 0.137) of Office 4. Moreover, similarity ratings between sections A (*t*_10_ = 0.67, *P* = 0.517) and section E (*t*_10_ = 0.30, *P* = 0.774) of Office 3 and either Office 4 or Office 5 did not differ anymore significantly. However, section E (*t*_10_ = 4.10, *P* = 0.010) of Office 4 was still rated unequally between Office 1 and Office 2. Due to a lack of further possible changes and the comparable ratings of the offices as whole (Office 1 vs. either Office 4 or Office 5: *t*_10_ = 0.68, *P* = 0.512; Office 2 vs. either Office 4 or Office 5: *t*_10_ = 0.65, *P* = 0.528; Supplementary Fig. 8b), we decided to keep this section as it was.

As expected, no significant differences were found for the five offices (Supplemental Table 1) in regard their valence (Pilot Study 1: *F*_4,60_ = 0.97, *P* = 0.430, partial η^2^ = 0.061; Pilot Study 2: *F*_4,40_ = 0.99, *P* = 0.426, partial η^2^ = 0.090) as well as their arousal (Pilot Study 1: *F*_4,60_ = 0.23, *P* = 0.922, partial η^2^ = 0.015; Pilot Study 2: *F*_4,40_ = 0.53, *P* = 0.717, partial η^2^ = 0.050).


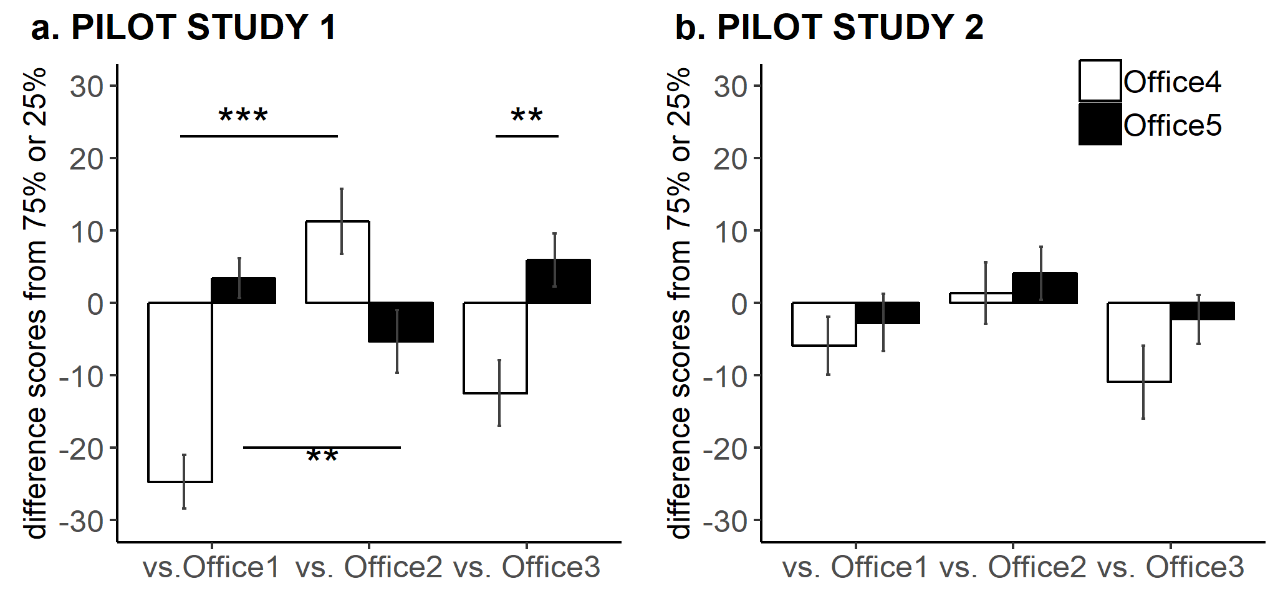


**Supplementary Fig. 8**. Differential scores for the similarity ratings. Participants rated the similarity between Office 1 and Office 4 or Office 5 as well as between Office 2 and Office 4 or Office 5. Importantly, Office 4 should be 75% similar to Office 1 and therefore we subtracted 75 from the similarity ratings between Office 1 and Office 4, parallel Office 5 should be 25% similar to Office 1 and therefore we subtracted 25 from the similarity ratings between Office 1 and Office 5. The same was calculated for Office 2, but the other way around. A score of 0 indicates the perfect proportion of similarity between Office 1 or Office 2 and the new offices.

|  | **Office 1** | **Office 5** | **Office 3** | **Office 4** | **Office 2** |
| --- | --- | --- | --- | --- | --- |
|  |  | **75% from Office 1 and 25% from Office 2** | **50% from Office 1 and 50% from Office 2** | **25% from Office 1 and 75% from Office 2** |  |
| **Pilot Study 1** |  |  |  |  |  |
| Valence Ratings (*SD*) | 47.19 (13.16) | 48.75 (11.18) | 54.00 (14.28) | 51.25 (12.18) | 52.19 (16.93) |
| Arousal Rating (*SD*) | 10.94 (10.36) | 11.88 (15.48) | 11.56 (12.48) | 10.31 (12.31) | 11.56 (14.23) |
|  |  |  |  |  |  |
| **Pilot Study 2** |  |  |  |  |  |
| Valence Ratings (*SD*) | 61.36 (14.85) | 61.82 (11.46) | 58.64 (12.67) | 55.91 (8.01) | 56.82 (10.55) |
| Arousal Ratings (*SD*) | 12.73 (12.72) | 11.82 (13.28) | 13.64 (13.62) | 12.27 (14.38) | 12.73 (14.21) |

**Reference**

1. Grillon C: **Startle reactivity and anxiety disorders: Aversive conditioning, context and neurobiology.** *Biological Psychiatry* 2002, **56**:958-975.

2. Andreatta M, Leombruni E, Glotzbach-Schoon E, Pauli P, Mühlberger A: **Generalization of contextual fear in humans**. *Behavior Therapy* 2015, **46**:583-596.
